# Periocular basal cell carcinoma results and surgical outcome during a 5-year period in a larger Danish population

**DOI:** 10.1186/s12886-022-02494-9

**Published:** 2022-06-28

**Authors:** Sveina Björk Karlsdóttir, Simon Johannessen, Nikolaj Carsting Bjerrum, Ulrik Frydkjær-Olsen, Søren Leer Blindbæk, Flemming Møller, Camilla Wellejus

**Affiliations:** Department of Ophthalmology, Sygehus Lillebaelt, 7100 Vejle, Denmark

**Keywords:** Surgery, Basal Cell Carcinoma, Biopsy, Pathology, Histology, Eyelids, Skin Neoplasms

## Abstract

**Background:**

To report tumour pathology, surgical procedure, complication rates and overall outcome of periocular basal cell carcinoma (BCC) in the Department of Ophthalmology at Sygehus Lillebaelt, Southern Denmark Region over a 5-year period.

**Methods:**

Medical records for all patients who underwent surgery for periocular BCC between January 2016 and December 2020 were reviewed. All tumours were excised with a 3 mm margin beyond the clinically apparent delimitation of the tumour and analysed by frozen section histological examination. Paraffin sections were subsequently examined for a final histopathological diagnosis. Patient age, gender, date of resection, former cancer history, referring unit and follow-up time were recorded. Furthermore, histological subtypes identified from biopsy and resection, lesion location, lesion diameter, free margin after the first operation, lacrimal punctum involvement, reconstructive techniques and complications were also recorded.

**Results:**

A total of 242 surgical excisions from 237 patients were recorded. The mean age was 69.7 ± 12.6 with women significantly predominant compared to men (1.8:1, *p* < 0.0001, binomial test). The mean tumour diameter was 4.29 mm (range 0.5–20 mm). The most common location and histological subtype was the lower eyelid and nodular BCC respectively (64.9% and 74.0% of cases). In 17.4% of the patients, the initial resection margin on the frozen section histology was not free of tumour cells and the risk was significantly greater for BCC subtypes considered aggressive in terms of growth pattern (morphea form, infiltrative and micronodular features) as compared to non-aggressive BCC subtypes (nodular and superficial) (*p* = 0.002, *X*^2^). In 239 (98.8%) of the patients, the BCC was found to be radically removed after final histopathological examination.

The sensitivity of identification of aggressive subtypes of periocular BCC in biopsies was 47.7%. No recurrences were found during the 5-year period.

**Conclusion:**

This study demonstrated a tendency towards more women than men being diagnosed with periocular BCC. The initial biopsy performed for all patients underestimated the aggressiveness of BCC in almost half of the cases while aggressive BCC subtypes were more likely to need further resection after frozen section compared to non-aggressive subtypes.

## Background

Basal cell carcinoma (BCC) is a non-melanoma skin cancer type and the most common type of skin cancer in Denmark [1, 2]. According to data from The Danish Cancer Registry, 14,657 new cases were registered in 2019 [3]. The overall incidence of BCC in Denmark is generally similar in both women and men and increases by age [1, 4]. Hence, with rising life expectancy, the number of patients diagnosed with BCC and in need of treatment is expected to increase in the years to come [5].

Approximately 80% of all BCC cases are diagnosed within the cervicofacial area [5, 6]. Even though most periocular tumours are of a benign nature [7], BCC is the most frequent malignant periocular tumour and 10–16% of all BCCs are diagnosed on the eyelids [8, 9]. Various subtypes of BCC have been described in the literature. The most common histological subtypes are nodular and superficial BCC which tend to be less aggressive. The morphea form, infiltrative and micronodular subtypes are rare but more aggressive in nature due to a higher rate of deep and irregular infiltration, which can result in incomplete excision and a higher risk of recurrence [10]. Even though the majority of BCCs can be classified into specific subtypes, it is not unusual to have a mixed pattern represented [11, 12].

BCC seldom metastasises [13] but the growth pattern may be highly destructive and cause severe tissue disfigurement leading to significant morbidity, especially when located periocularly. Surgical excision of BCC with sufficient margins in the periocular region can be challenging. Preservation of both the eyelid function and skin to protect the vision is crucial for optimal results. In addition to this, achieving an acceptable cosmetic result is another challenge to overcome [14].

The main objective of the present study was to review the results from surgical excision of periocular BCC over a 5-year-period at the Department of Ophthalmology, Vejle Hospital, Southern Denmark Region. Tumour pathology, surgical procedure, complication rates and overall outcome were reviewed. To the best of our knowledge, such data, which includes information relevant to both patients and clinicians, has not previously been reported from a Scandinavian population.

## Methods

### Setting

A retrospective case series study from the Department of Ophthalmology, Vejle Hospital, Southern Denmark Region.

### Participants

Medical records of 237 patients with 242 tumours were reviewed during a 5-year period between 1^st^ January 2016 and 31^st^ December 2020. All patients were diagnosed and surgically treated for periocular BCC at the Department of Ophthalmology, Sygehus Lillebaelt, Southern Denmark Region. Only patients with histologically confirmed BCC on biopsies or the final resection were included. Date of resection, patient age at resection, gender, former cancer history, referring unit and time at follow-up were recorded. Furthermore, histological subtype identified from biopsies and after resection, lesion location and diameter, the proportion with free margins after first resection, lacrimal punctum involvement, reconstructive techniques and complications were recorded.

Nodular and superficial BCC subtypes were categorised as non-aggressive while infiltrative, morphea and micronodular subtypes were categorised as aggressive. In the case of a mixed pattern of aggressive and non-aggressive BCC subtypes, the tumour was categorised as aggressive.

### Surgical procedure

Tumours were resected with at least a 3 mm margin evaluated macroscopically and orientation was marked with a suture. The excision specimen was histologically examined by frozen section. If the tumour exceeded any margins, further resection was performed until radicality was achieved. A suitable method for reconstruction was determined by the surgeon and depended on the resection size and location. Direct closure was used when possible and in the remaining cases, a standard reconstructive technique was performed. The final histological assessment of the tumour was based on paraffin-embedded section.

## Results

A total of 237 patients were surgically treated for 242 instances of histopathologically proven periocular BCC. All patients were Caucasian. Women were significantly predominant compared to men with a ratio of 1.8:1 (154 females and 88 males, *p* < 0.0001, binomial test) with the mean age for women and men at 70.0 ± 13.2 years and 70.9 ± 11.8 years respectively (Table 1). Fourteen patients had died, due to reasons unrelated to BCC, at the time of the case review. The mean follow-up was 72 days (range 1 to 859 days). Sixty-one patients (25%) had a positive BCC biopsy prior to referral and 41 patients (27 women and 14 men) had a history of previous skin cancers. The vast majority of periocular BCC had not previously been diagnosed (98.8%) while 1.2% represented recurrence of BCC that had previously been surgically removed. The referring units and the number of patients included from each referring unit are listed in Table 2.

**Table 1:** Number of periocular BCC by gender and year

	All	Women	Men	Ratio (Women:Men)
2016–2021	242	154	88	1.8:1*
2016	26	16	10	1.6:1
2017	43	29	14	2.1:1*
2018	53	34	19	1.8:1*
2019	59	34	25	1.4:1*
2020	61	41	20	2.1:1*

^*^p < 0.05 (binomial test)

**Table 2:** Referring unit

	n (%)
	All (*n* = 242)
Ophthalmologist, private practicing	142 (58.7)
General Practitioner	4 (1.7)
Dermatologist, private practicing	40 (16.5)
Interdisciplinary conference^a^	36 (14.9)
Internally	14 (5.8)
Other Hospitals	6 (2.5)

^a^ With oncology and/or plastic surgery department

The tumour size (largest diameter) was recorded in 165 patients with a mean of 4.21 ± 3.31 mm for women (*n* = 108) and 4.49 ± 3.75 mm for men (*n* = 57). Right and left side was involved in 124 and 118 of the cases respectively. The lower eyelid was the most frequent location (64.9%) whereas the medial canthus was the region with the highest rate of non-free margin at first resection (36.4%). Table 3 shows the periocular anatomic tumour location and the distribution of cases where the excision margin was not free of tumour cells.

**Table 3:** Tumour location

	n (%)	
	All (*n* = 242)	Margin not free on frozen section^a^ (*n* = 42)
Inferior palpebrae	157 (64.9)	26 (61.9)
Medial canthus	33 (13.6)	12 (28.6)
Lateral canthus	4 (1.7)	0
Superior palpebrae	25 (10.3)	4 (9.5)
Nasal spine	20 (8.3)	0
Cheek	1 (0.4)	0
Eyebrow	2 (0.8)	0

^a^ Distribution of anatomic location when the margin on the initial frozen section specimen was not free of tumour cells

A biopsy of all 242 tumours was performed prior to excision. In three cases, the initial biopsy showed growth of another type of cancer although the subsequent histological assessment revealed a BCC. The final histological assessment revealed 11 specimens with no residual tumour after biopsy and a cancer type other than BCC (planocellular carcinoma or basosquamous carcinoma) was found in three cases. The distribution of histological subtypes in the biopsies and the final histological assessment respectively is shown in Table 4. Sensitivity and specificity of the biopsy to differentiate between aggressive and non-aggressive periocular BBC was 47.7% and 93.9% respectively (Table 5).

**Table 4:** Histological subtypes

	All (*n* = 242)	All (*n* = 242)
	n (%)	n (%)
	**Biopsy**	**Histological (paraffin-embedded section)**
Non-aggressive type n (%)	206 (85.1)	182 (75.2)
Nodular	199 (96.6)	178 (97.8)
Superficial	7 (3.4)	4 (2.2)
Aggressive type n (%)	33 (13.6)	46 (19.0)
Infiltrative	25 (75.6)	39 (84.8)
Micronodular	6 (18.2)	7 (15.2)
Morphea	2 (6.1)	0
Other^a^ n (%)	3 (1.2)	14 (57.9)
Other types^a^	3 (1.2)	3 (21.4)
No residual tumour^b^:	0	11 (78.6)

^a ^Planocellular Carcinoma or Basosquamous Carcinoma

^b ^No residual tumour was found during the final histological assessment

**Table 5:** Biopsy sensitivity and specificity

Biopsy versus histology	Aggressive (histology)	Non-aggressive (histology)	All
Aggressive (biopsy)	21	11	32
Non-aggressive (biopsy)	23	170	193
All	44	181	

Sensitivity: 21/44: 47.7%, specificity: 170/181: 93.9%

Of the 242 tumours, 17 were excluded in the calculations. In three of the biopsies, a basosqumous carcinoma

was found (see Table 4). In the final histological assessment, no rest tumour was found in 11 cases and tumour cells other than BCC were noted in three cases (see Table 4)

Patients with an aggressive BCC subtype (*n* = 46) had a significantly higher rate of incomplete primary excision (30.4%) compared with the non-aggressive (*n* = 182) BCC subtypes (14.8%) (*p* = 0,002, *X*^2^). The final histological assessment (paraffin-embedded section) additionally revealed three tumours with non-free margins: one aggressive and two non-aggressive periocular BCCs. Table 6 demonstrates the proportion of free margins at primary excision between aggressive and non-aggressive BCC subtypes.

**Table 6:** Free margin after first resection

	Yes n (%)	No n (%)
	All 200 (82,6)	All 42 (17.4)
Non-aggressive n (%)	155 (77.5)	27 (14.8)
Aggressive n (%)	32 (16.0)	14 (30.4)
Other^a^ n (%)	13 (6.5)	1 (0.07)

^a ^Planocellular carcinoma

Direct closure was performed in 34.3% of cases whereas 65.7% required more advanced reconstructive techniques. For patients diagnosed with an aggressive BCC subtype, there was a significantly greater need for a more advanced reconstruction technique other than direct closure (*X*^2^, *p* = 0.004, Table 7).

**Table 7:** Type of operations

	Direct closure n (%): All 83 (34.3)	More complicated reconstruction n (%): All 159 (65.7)
Aggressive n (%)	8 (9.6)	38 (23.9)
Non-aggressive n (%)	68 (82)	114 (71.7)
Other n (%)	7 (8.4)	7 (4,4)

Forty-eight patients (19.8%) experienced one or more complications during follow-up (Table 8). The most common complication after reconstruction of the eyelids was ectropion. However, only one patient needed surgical correction for ectropion. No graft or flap-failures were observed. Symblepharon formation developed after reconstruction with a modified Hughes flap and needed revision that was completed without graft failure. Two patients needed hospitalisation due to bleeding; both were on non-vitamin K-oral anticoagulant treatment.

**Table 8:** Complications

	All (*n* = 242)
Non n (%)	180 (74.4)
All n (%)	48 (19.8)
Ectropion	12 (4.9)
Infection	9 (3.7)
Corneal abrasion	6 (2.5)
Suture granuloma	3
Other suture complication	5
Pain	3 (1.2)
Oedema (inj. Kenalog)	1 (0.4)
Need for revision of skin/conjunctiva	8 (3.3)
Bleeding and need of hospitalization	2 (8.3)
Epifora	5 (2.1)
Graft/flap failure	0
Lost to follow-up n (%)	14 (5.8)

Some patients had more than one complication

Involvement of the proximal lacrimal apparatus was seen in twenty-eight of the surgical procedures (11.6%).

## Discussion

To our knowledge, this retrospective study is the first to address characteristics and treatment outcomes in periocular BCC in a large Scandinavian population. Mean ages were comparable between women and men, and consistent with previously published data [15, 16]. In our study, periocular BCC was found almost twice as frequently in women as in men. Although this tendency has previously been reported in younger patients [1, 17–19], it is in contrast to most other studies [20, 21] where an even gender distribution or a predominance in men has been demonstrated [22, 23]. Likewise, a higher prevalence of BCC outside the periocular region has been found in men compared to women for patients aged over 60 whereas there was no difference in prevalence for individuals below the age of 60 [1]. However, there are reports of a rapid and disproportionate increase in BCC in younger women [19, 24] and a recent study from Iceland revealed the incidence of BCC to be significantly higher in women compared to men [25]. It has been speculated that this tendency may be explained by the dissimilar use of solariums between men and women [26].

In accordance with previous studies [24, 27, 28], we observed the lower eyelid to be the most common anatomical location and nodular BCC was the most common subtype. Furthermore, and also in accordance with previous studies [29, 30], we found that an incomplete excision was more common for the aggressive subtypes (30.4%, Table 4) compared to the non-aggressive subtypes (14.8%). Likewise, tumour location was also important since incomplete tumour excision was more frequently observed in the medial canthal region (36%, Table 3) [31]. This may be because of a narrower safety margin to the tumour in an attempt to preserve vital medial canthal structures.

Surprisingly, we found that the initial biopsy underestimated the aggressiveness of the tumour in 47.7% of cases compared to the final complete histological examination. In a study by Wolberink et al*.*, 31% of BCCs were found to be incorrectly classified by the initial biopsy [32]. Better results were found by Haws et al*.* who reported a discrepancy of 18% in histological subtyping between biopsy and the final examination [33]. A randomised controlled trial found better accuracy (73.8%) when taking punch biopsies from two sites compared to one [34]. However, inaccuracy between the biopsy and final histopathological diagnosis regardless of the test method warrants better preoperative evaluation of tumour delineation.

Surgical excision with a 3 mm safety margin was performed in the present study. The rationale for this procedure was that some BCC subtypes tend to infiltrate the surrounding area in a three-dimensional pattern, which leads to subclinical tumour spread [21]. We found that 17.4% of periocular BCCs had non-free margins on the first resection. This is numerically slightly lower than previously reported but may however vary greatly depending on the chosen safety margin to the macroscopic tumour delimitation [20, 31].

Newer techniques are available which allow for a more detailed examination of the skin. Conventional optical coherence tomography (OCT) may have a role in the identification and subtyping of BCC and possibly limit the margins for safe excision, which may decrease the need for more advanced surgery as well as produce better cosmetic results for the patients [35].

Patients discharged from the department were recommended a yearly control for 5 years by an ophthalmologist in primary care. Within the 5-year period of our study, no recurrences were identified. These findings are in agreement with a study by Hsuan et al*.* which reported no recurrences after excision of nodular BCC with a 2 mm safety margin after five years of follow-up [36]. A recurrence rate of 5.6% overall was reported in a large retrospective study with a 16-year follow-up from Spain [20].

The rate of surgical complications in this present study was comparable to previous studies [20, 37] even though 65.7% of patients underwent advanced reconstructive surgery.

## Conclusions

In conclusion, we found a tendency towards more women compared to men being diagnosed with BCC. Furthermore, the initial biopsy underestimated the aggressiveness of the tumor in almost half of the cases increasing the risk of a false-negative histopathological diagnose, which may delay the surgical treatment. Accordingly, in our setting more attention should be paid to the biopsy procedure. In 17.4% of the excised tumors, the margin of the 3 mm safety zone was not free of tumor cells, thus excising periocular BCC´s without histologically examinations by frozen section bares a potential risk of non-radical removal. However, no recurrence of BCC with a 3 mm safety margin was noted at the end of the study although the observation time was limited.

## Data Availability

The data are availbale from the corresponding author upon reasonable request.

## Abbreviation

BCC: Basal Cell Carcinoma
